# Impact of additional resection on new ischemic lesions and their clinical relevance after intraoperative 3 Tesla MRI in neuro-oncological surgery

**DOI:** 10.1007/s10143-020-01399-9

**Published:** 2020-09-30

**Authors:** Stefanos Voglis, Timothy Müller, Christiaan H. B. van Niftrik, Lazar Tosic, Marian Christoph Neidert, Luca Regli, Oliver Bozinov

**Affiliations:** 1 Department of Neurosurgery and Clinical Neuroscience Center, University Hospital and University of Zurich, Frauenklinikstrasse 10, 8091 Zurich, Switzerland; 2 Department of Neurosurgery, Kantonsspital St. Gallen, Medical School St. Gallen, Rorschacher Strasse 95, 9007 St. Gallen, Switzerland

**Keywords:** Intraoperative MRI, Diffusion-weighted imaging, DWI, Infarcts, Ischemia, Brain tumor, Neurological outcome

## Abstract

Intraoperative MRI (ioMRI) has become a frequently used tool to improve maximum safe resection in brain tumor surgery. The usability of intraoperatively acquired diffusion-weighted imaging sequences to predict the extent and clinical relevance of new infarcts has not yet been studied. Furthermore, the question of whether more aggressive surgery after ioMRI leads to more or larger infarcts is of crucial interest for the surgeons’ operative strategy. Retrospective single-center analysis of a prospective registry of procedures from 2013 to 2019 with ioMRI was used. Infarct volumes in ioMRI/poMRI, lesion localization, mRS, and NIHSS were analyzed for each case. A total of 177 individual operations (60% male, mean age 45.5 years old) met the inclusion criteria. In 61% of the procedures, additional resection was performed after ioMRI, which resulted in a significantly higher number of new ischemic lesions postoperatively (*p* < .001). The development of new or enlarged ischemic areas upon additional resection could also be shown volumetrically (mean volume in ioMRI 0.39 cm^3^ vs. poMRI 2.97 cm^3^; *p* < .001). Despite the surgically induced new infarcts, mRS and NIHSS did not worsen significantly in cases with additional resection. Additionally, new perilesional ischemia in eloquently located tumors was not associated with an impaired neurological outcome. Additional resection after ioMRI leads to new or enlarged ischemic areas. However, these new infarcts do not necessarily result in an impaired neurological outcome, even when in eloquent brain areas.

**Electronic supplementary material:**

The online version of this article (10.1007/s10143-020-01399-9) contains supplementary material, which is available to authorized users.

## Introduction

Intraoperative MRI during cranial tumor surgery has become a supplementary standard of care in many neurosurgical institutions for a variety of neurosurgical procedures [4, 12, 22, 23]. The intraoperative images are of great value to provide the surgeon with an updated dataset for navigation, along with intraoperative resection control and early screening for complications such as ischemic complications. Several studies have reported the utility of intraoperative MRI (ioMRI) to maximize safe resection, which, in turn, is linked to prolonged overall survival in patients with, for example, low- and high-grade gliomas [7, 13, 22, 24]. Increasing experience has led to more sophisticated protocols and guidelines, which have improved the safety [1, 19] and the robustness and quality of ioMRI sequences. This accounts not only for routinely used methods but also for advanced imaging methods such as diffusion-weighted imaging (DWI) [17, 25]. DWI sequences acquired during surgery are used as an important tool for the early detection of ischemic brain injuries that might cause new neurological deficits for the patient. Therefore, these sequences potentially influence the surgeon’s decision to continue the resection or not.

Despite the evident advantages of ioMRI, little is known about the influence of DWI sequences on surgical strategy and aggressiveness in terms of resection control and their implications for potential new postoperative deficits. The aim of this study was to characterize the presence and extent of new ischemic lesions in ioMRI. Additionally, we were interested in the question of whether a continuation of resection after intraoperative resection control with MRI might lead to more or enlarged postoperative infarcts.

## Methods

### Patient selection

**Fig. 1:**
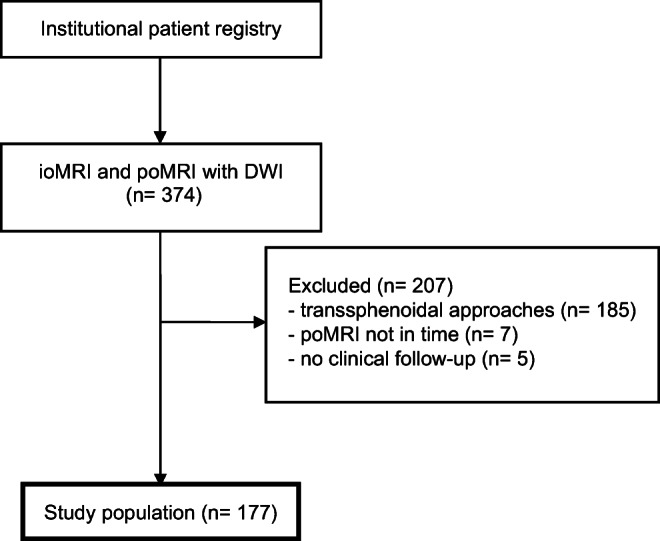
Flowchart of patient inclusion criteria. ioMRI intraoperative MRI, poMRI postoperative MRI, DWI diffusion-weighted imaging

All consecutive patients who underwent neurosurgical procedures using ioMRI with acquired DWI sequences between 01/2013 and 01/2019 at our department were included. Transsphenoidal procedures with ioMRI were excluded because the ioMRI protocol changed during the observation period, and acquisition of DWI sequences in these procedures was omitted; ioMRI was only used inconsistently. Regularly, patients in which resection was extended after ioMRI received postoperative MRI (poMRI) within 72 h. However, in singular cases, no early poMRI was done. To ensure comparability of new DWI restrictions, cases in which surgical resection was expanded after ioMRI poMRI was not conducted within 14 days after surgery were excluded (Fig. 1).

### Intraoperative MRI

Our institution uses a 2-room intraoperative MRI suite concept with a 3 Tesla (T) high-field MRI (Siemens 3 T Skyra VD13, Siemens Healthineers, Erlangen, Germany) and a NORAS 8 channel head coil (NORAS MRI products GmbH, Hoechberg, Germany). The safety and quality of intraoperatively acquired MRI sequences were ensured by using a previously published institutional default ioMRI checklist [25].

### Data acquisition

Data points were extracted from our departmental prospectively recorded patient registry, which includes a standardized dataset for all neurosurgical procedures since 2013 [20]. The registry was approved by the local ethical review board (“Kantonale Ethikkommission Zürich,” identifier PB-2017-00093), registered at clinicaltrials.gov (NCT01628406), and individual patients’ consent was waived. All data were collected by neurosurgeons at hospital admission and discharge. At discharge, all data entries were validated by the respective surgeon. The modified Rankin Scale [26] (mRS) is used as a general performance scale, and the National Institute of Health Stroke Scale [11] (NIHSS) is used as a neurological outcome scale. For every new team member, it is obligatory to complete an online introductory teaching course and exam to ensure the correct usage of the clinical scores. Missing data for any timepoint were added by retrospective chart review.

### Infarct determination and volumetric analysis

**Fig. 2:**
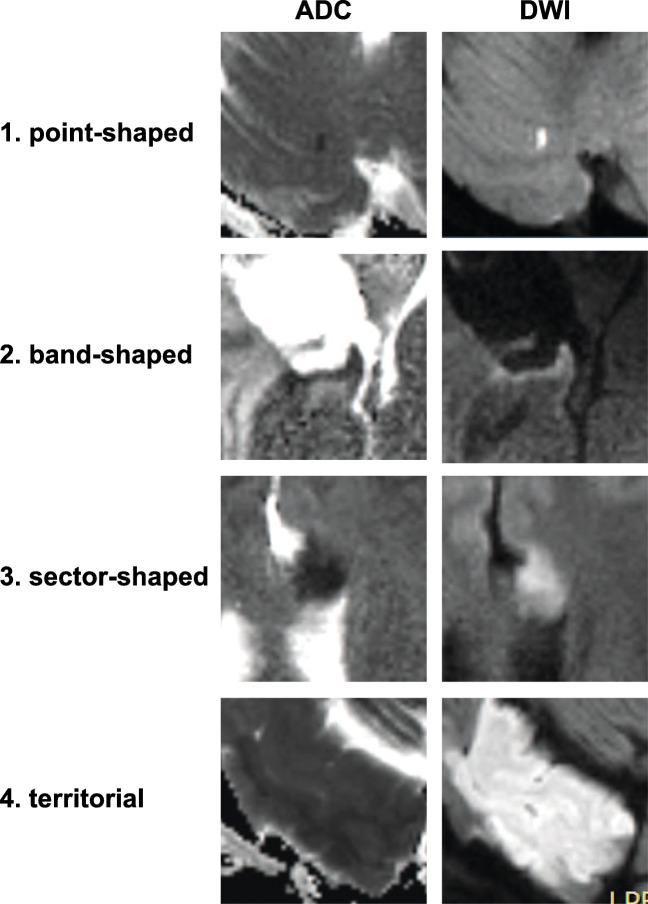
Examples of different infarct categories with their corresponding ADC and DWI images. 1. Point-shaped infarct; 2. thin band-shaped infarct at resection cavity; 3. sector-shaped infarct with involvement of deeper parts of the parenchyma; 4. territorial infarct. ADC apparent diffusion coefficient, DWI diffusion-weighted imaging

Infarcts were determined using DWI and apparent diffusion coefficient (ADC) image series for each case. Susceptibility weighted imaging sequences were used to rule out artificial DWI restrictions caused by microhemorrhage, especially at the resection cavity. Based on the extent and localization of the DWI restriction, infarcts were grouped into 4 classes in ascending order: 1. point-shaped infarcts, 2. band-shaped infarcts at the resection cavity, 3. sector-shaped infarcts that involve deeper parts of the parenchyma, and 4. territorial infarcts (Fig. 2). If more than one infarct class was present, only the highest class was registered, whereas for volumetric measurements, all infarct areas were summed. Volumetric analysis of infarct volumes from DWI sequences was performed using iPlan Net® (Brainlab AG, Munich, Germany). The relative change in infarct volume was calculated by subtracting intraoperative from postoperative infarct volumes.

### Statistical analysis

All data processing steps and statistical analyses were performed using R Studio (Version 1.1.463, R Studio Inc.) with open-source libraries. *p* values < .05 were considered statistically significant. The statistical tests used are indicated in the figure captions or the main text. There were no missing data for the variables included in the analysis.

## Results

### Clinical characteristics

The inclusion criteria resulted in a cohort of 177 surgical procedures (Fig. 1) in 155 individual patients. The mean age was 45.5 years (± 16.8 SD; 3–76), and 60% of the cases involved male patients. The mean age of patients did not differ significantly between patients with and without new infarcts on poMRI (Mann-Whitney *U* test, *p* = .29, Table 1). Sixty percent of the cases were first operations, whereas the remaining cases involved a previous operation on the lesion (Table 1). This difference was not associated with a higher number of new infarcts on poMRI (Pearson’s chi-squared test, *p* = .2, Table 1). Histopathological entities are listed in Table 1, with WHO grade III (*n* = 57) and IV (*n* = 51) gliomas (total *n* = 145) being the most frequent. Metastases (*n* = 9) derived most frequently from lung carcinoma (*n* = 6, Online Resource 1 – Suppl. Table 1). Other histopathological entities included epidermoid cysts (*n* = 3), central neurocytoma (*n* = 2), schwannoma (*n* = 2), and others (Online Resource 1 – Suppl. Table 1).

**Table 1:** Patient characteristics

Characteristics	No.	Distribution	poMRI new infarct compared with ioMRI		*p* value
			Yes	No	
Age		45.5 (± 16.8; 3–76)	46.9 (± 15.4; 9–74)	43.3 (± 18.7; 3–76)	.289
Sex					
Male	106	60%	68 (64%)	38 (36%)	.208
Female	71	40%	38 (53.5%)	33 (46.5%)	
Reoperation	71	40%	38 (53.5%)	33 (46.5%)	.208
Histology					
Glioma	145	82%	94 (65%)	51 (35%)	.006
Others	23	13%	7 (30%)	16 (70%)	
Metastases	9	5%	5 (56%)	4 (44%)	
WHO classification					
I	6	4%	3 (50%)	3 (50%)	.6
II	31	21%	20 (64.5%)	11 (35.5%)	
III	57	39%	40 (70%)	17 (30%)	
IV	51	35%	31 (61%)	20 (39%)	
Anatomical localization					
Frontal	64	36%	36 (56%)	28 (44%)	.13
Perirolandic	22	12%	12 (54.5%)	10 (45.5%)	
Temporal	17	10%	12 (71%)	5 (29%)	
Insular	15	8%	13 (87%)	2 (13%)	
Limbic	15	8%	12 (80%)	3 (20%)	
Multifocal	10	6%	5 (50%)	5 (50%)	
Lateralization*					
Right	98	55%	61 (62%)	37 (38%)	.6
Left	74	41%	43 (58%)	31 (42%)	
Midline	4	2%	2 (5%)	2 (5%)	
Resection after ioMRI					
Yes	108	61%	89 (82%)	19 (18%)	< .001
No	69	39%	17 (25%)	52 (75%)	

Study population characteristics. Significance levels were calculated using Pearson’s chi-squared, Fisher’s exact, or Mann-Whitney *U* test, as appropriate

*ioMRI* intraoperative MRI, *poMRI* postoperative MRI, *No.* number

*One case with bilateral lesions

### Determinants of new DWI lesions on poMRI

The frequency of new postoperative infarcts differed between histological tumor entities: gliomas and metastases showed new infarcts in 65% and 56%, respectively, compared with only 30% for other entities (Fisher’s exact test, *p* = .006, Table 1). WHO classification, however, did not seem to be associated with new infarcts in poMRI (Fisher’s exact test, *p* = .6, Table 1). Looking at the localization of the operated lesions within the brain, the frontal lobe was the most frequently affected lobe (36%), followed by the perirolandic region (12%) and the temporal lobe (10%). However, the occurrence of new postoperative ischemic lesions was not linked to a distinct anatomical localization of the resected lesion (Fisher’s exact test, *p* = .13). Intraoperative neuromonitoring (ioNM) was used in 60.5% of resections and declined at least at one point during surgery in 21.5%. Resection was stopped according to operative report because of ioNM worsening in 17% of the cases with ioNM. However, usage, decline, and stop because of decline were not associated with new infarcts in poMRI (Online Resource 1 – Suppl. Table 2).

In 61% (*n* = 108) of the cases, surgical resection of the lesion was continued after ioMRI. This resulted in a significantly higher number of postoperative ischemic lesions compared with cases without additional resection after ioMRI (Pearson’s chi-squared test, *p* < .001). Nonetheless, in cases without additional resection, the absence of DWI lesions in ioMRI vs. poMRI showed a specificity of 78.4%. Across the whole study group, the presence of an intraoperative DWI restriction could be confirmed on poMRI in the majority of cases (83.7%, positive predictive value).

### Infarct characteristics and quantification

**Fig. 3:**
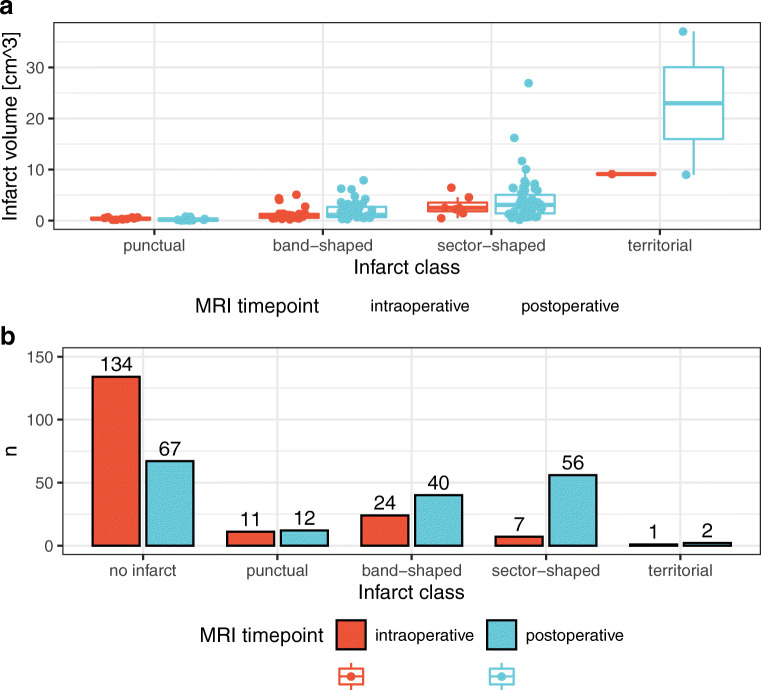
**a** Volumetric and morphological characteristics of the new infarcts. Infarct volumes in cm^3^ of the different infarct categories (Fig. 1) for intraoperative and postoperative MRI (Wilcoxon signed-rank test *p* > .5 for each infarct class). **b** Number of demarcated new infarcts of each morphological infarct category (Fig. 1) in intraoperative (red) and postoperative (blue) MRI

Classifying the infarcts regarding their shape (Fig. 2), the distribution of infarct volumes among the different classes is shown in Fig. 3a for ioMRI and poMRI. Median infarct volumes increased throughout the different infarct classes, and there was an increased mean infarct volume in poMRI compared with ioMRI (Fig. 3a): the increase was slight in band-shaped (1.97 ± 1.81 vs. 1.39 ± 1.32 cm^3^) and more pronounced in sector-shaped (4.01 ± 4.32 vs. 2.89 ± 2.0 cm^3^) and territorial infarctions (23.01 ± 19.8 vs. 9.12 cm^3^). However, statistical comparison (Wilcoxon signed-rank test) showed no significant difference between volumes of different infarct classes in intra- vs. poMRI. Figure 3b shows the absolute numbers of the different infarct classes for ioMRI and poMRI. Fewer cases had no infarct demarcation (*n* = 67) in poMRI than in ioMRI (*n* = 134). A total of 94% (*n* = 63) of cases with new infarct on poMRI underwent additional resection after ioMRI (vs. 6%, *n* = 4 without additional resection). This increased number of new postoperative infarcts resulted mainly from new band-shaped and sector-shaped infarcts adjacent to the resection cavity, whereas point-shaped and territorial ischemic lesions showed comparable numbers intra- and postoperatively (Fig. 3b).

### Additional resection upon ioMRI

In our case series, a significantly increased overall infarct volume occurred in cases with continuation of resection after ioMRI (Pearson’s chi-squared test, *p* < .001).

**Fig. 4:**
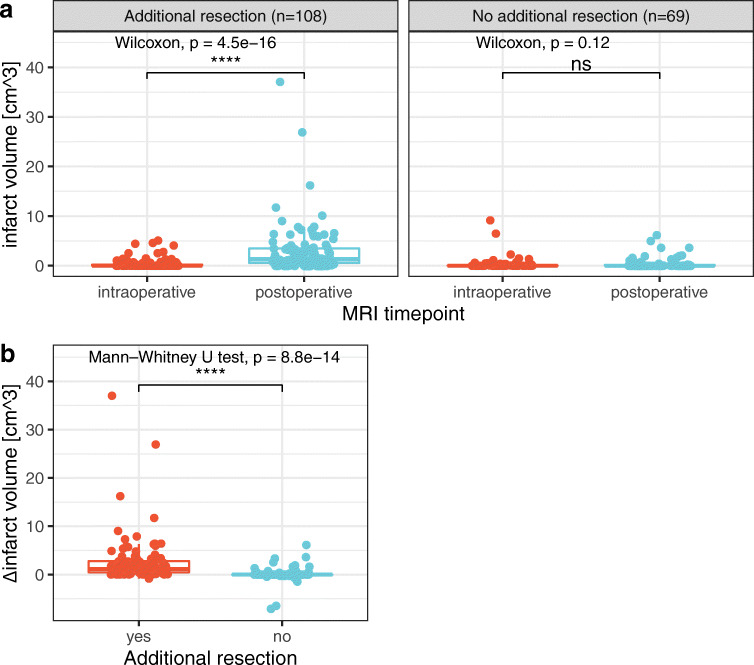
**a** Comparison of absolute infarct volumes (cm^3^) in intraoperative (red) and postoperative (blue) MRI (Wilcoxon signed-rank test) for cases with (left plot) and without additional resection (right plot) after intraoperative MRI. **b** Comparison of relative infarct volume change (postoperative–intraoperative MRI) of cases with (red) and without (blue) additional resection upon intraoperative MRI (Mann-Whitney *U* test). Δ delta, ns not significant

Comparing the infarct volumes regarding additional resection after ioMRI, the cases with additional resection showed significantly increased postoperative infarct volumes (0.39 cm^3^ vs. 2.97 cm^3^ mean volume; Fig. 4a left plot, Wilcoxon signed-rank test, *p* < .001). When comparing the relative changes in infarct volumes between ioMRI and poMRI (Δ infarct volume, Fig. 4b), the enlarged ischemic lesions in cases with additional resection become more evident (Mann-Whitney *U* test, *p* < .001). In contrast, in cases where resection was finished after ioMRI, the absolute and relative ischemic volumes did not increase significantly (Fig. 4a right plot, b), although singular cases showed a slightly increased infarct volume in poMRI. The majority of increased infarct volumes resulted from entirely new infarcts in poMRI and less from enlarged or evolved preexisting ones (see Online Resource 2 – Suppl. Fig. 1 and Suppl. Fig. 2 for an illustrative example).

Moreover, the occurrence of a DWI lesion in ioMRI did not affect the proportion of continued resections (65.1% of cases with new DWI compared with 59.7% without new DWI restriction in ioMRI; Pearson’s chi-squared test *p* = .65), and the histopathological subdivision was similarly not associated with an altered proportion of continued resection after ioMRI (Fisher’s exact test, *p* = .08).

### Neurological and functional outcome upon new infarct demarcation

**Fig. 5:**
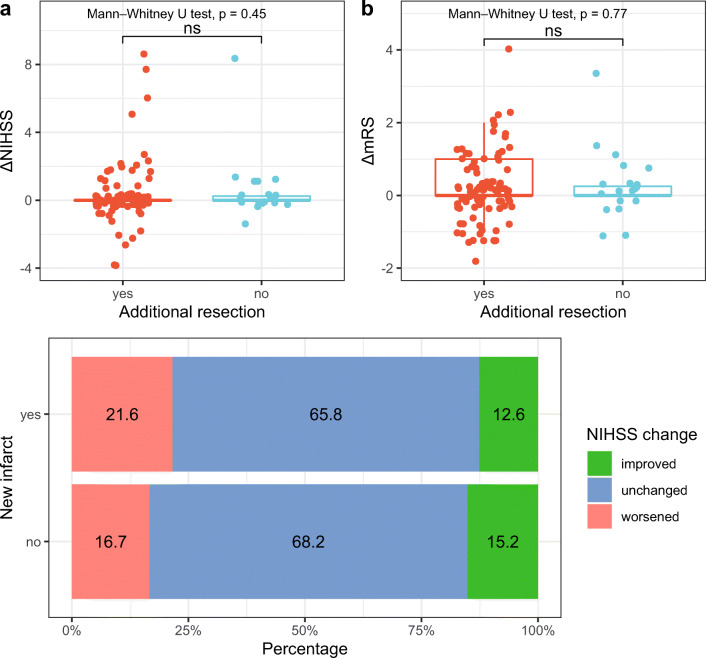
**a**, **b** Comparison of relative changes in NIHSS (**a**) and mRS (**b**) at discharge compared with admission for cases with (red) and without (blue) additional resection after intraoperative MRI; only cases with a new infarction on postoperative MRI are shown. **c** Stacked bar plots indicating the percentages of improved (green), unchanged (blue), or worsened NIHSS scores postoperatively for cases with (upper bar) and without (lower bar) new infarcts. ΔNIHSS delta National Institute of Health Stroke Scale, ΔmRS delta modified Rankin Scale, ns not significant

We compared the relative changes in NIHSS (Fig. 5a) and mRS (Fig. 5b) at discharge compared with admission for all cases with new infarction on poMRI. The score changes for cases with additional resection did not differ significantly from the cases without additional resection after ioMRI (Mann-Whitney *U* test, *p* > .05 for ΔNIHSS and ΔmRS). Looking at the percentages of cases with improved, unchanged, and worsened NIHSS after surgery, there was no significant difference between the group with and the group without new postoperative infarct (Pearson’s chi-squared test *p* = .7, Fig. 5c).

### Relevance of anatomic lesion localization for clinical impact of new infarcts

As new infarcts might only become clinically apparent if they are localized in certain brain areas, we identified the anatomical localization of the operated lesions and their lateralization (Table 1): Resections within the frontal lobe represented the main part of the patient population, followed by lesions involving the perirolandic region. Although there were several resections in the perirolandic region (12%, *n* = 22), which is considered a highly eloquent brain area, we detected no statistically significant difference in relative NIHSS change between cases with new ischemic areas and those without new infarcts (Table 1 and Online Resource 2 – Suppl. Fig. 3).

## Discussion

Intraoperative MRI has become a widely implemented tool in neurosurgical procedures to maximize safe resection for applications such as brain tumor surgery [4, 7, 13, 22]. Despite the improved quality of the intraoperatively acquired MRI sequences [17], little is known about the usability of intraoperatively acquired DWI sequences, their influence on surgical strategy, and the risk and clinical impact of new postoperative infarcts due to an additional resection after ioMRI.

In the current study, we showed that intraoperatively acquired DWI images with a 3T high-field MRI are of enough quality to early rule out or delineate new ischemic lesions.

The usage strategy of ioMRI remains inconsistent, as some consider it as a confirmation of complete tumor removal and resection should be considered finished by the surgeon before ioMRI is performed, others referring to the so called “staged volume” surgery, allowing a stepwise tumor resection by repeating ioMRI and thus increasing safety [9]. However, in many published series, additional resection is performed after acquisition of ioMRI [21], as was also the case in our study population.

Our data clearly show that an additional resection after ioMRI leads to an increased occurrence and volume of ischemic lesions on poMRI. These findings confirmed our expectations since resection after ioMRI is usually continued deeper and often more difficult to access or eloquently seated tumor remnants.

In the histopathological subgroup of gliomas (82% of the cases), the occurrence of new infarcts after additional resection was significantly higher than in other entities. Since the overall goal of maximum (safe) resection is of crucial interest for the long-term outcome of glioma surgery [13, 22, 24], this finding did not surprise us, as usage of intraoperative resection control might be prone to a more aggressive resection compared with cases without.

The differentiation between the first operation or reoperation on the respective cerebral lesion showed no differences regarding the occurrence of new infarcts. This might seem counterintuitive and in contrast with previous findings, which indicate that postoperative ischemic lesions occurred more frequently in recurrent glioma surgery compared with resections of primary gliomas [3]. However, the literature reports remain ambiguous, as another study concluded that recurrent glioma surgery was not associated with more infarcts [2]. In contrast to the cited studies, our patient cohort consists of a broader variety of histopathological entities and not all tumor reoperations might have received radiotherapy and developed postradiogenic tissue changes, which could potentially contribute to a higher rate of infarctions in recurrent surgery [3, 27]. Additionally, the proper use of ioMRI in recurrent glioma surgery might improve safe resection compared with recurrent surgeries without intraoperative image guidance.

To avoid vascular lesions, a combination of central tumor debulking using e.g. ultrasonic aspiration to provide sufficient working space and subsequent sub-pial resection to protect vasculature and normal brain tissue might be recommendable.

Previous studies indicated that increased peritumoral DWI restriction in poMRI is associated with new deficits in patients who underwent glioma surgery [3, 6]. However, our data indicate that new DWI restrictions due to extended resection after ioMRI are not necessarily clinically relevant in terms of impaired neurological outcome. As the current study did not look at the clinical relevance of general new infarcts after surgery but rather that of new infarcts caused by additional resection after ioMRI, our findings do not necessarily contradict the results of previous studies.

In our case series, we observed a few cases where no additional resection was performed after ioMRI but enlarged DWI restrictions were nevertheless detected on poMRI. Although DWI images were previously described to reach a high sensitivity and specificity to detect ischemic brain lesions [5], false-negative reports of DWI sequences are present. This phenomenon of false-negative or underestimated infarcts on MRI is not well described and poorly understood, especially in ioMRI [10, 15, 18] and also in hyperacute stroke imaging [8, 14–16]. Possible explanations might be that ioMRI occurs too early to detect hyperacute infarcts or that vasospasms contribute to delayed infarct development without further tissue manipulation by the surgeon. Further studies are necessary to evaluate the role of false-negative MRI with no DWI signal alteration in an intraoperative application and in general.

## Conclusion

The surgeon’s decision to continue resection after ioMRI bears the risk of additional and enlarged ischemic brain lesions. However, this study shows that these new infarcts do not necessarily result in an impaired neurological outcome, even if the operation occurs in eloquent brain areas.

## Electronic supplementary material

ESM 1 (PDF 129 kb).

ESM 2 (PDF 525 kb).

## Data Availability

From the corresponding author upon reasonable request.
