# Supplementary material for: Impact of additional resection on new ischemic lesions and their clinical relevance after intraoperative 3 Tesla MRI in neuro-oncological surgery
Source: Neurosurg Rev. 2020 Sep 30;44(4):2219–27. doi: 10.1007/s10143-020-01399-9 (PMC8338811; doi:10.1007/s10143-020-01399-9)
Supplement: Supplementary file 1 — (PDF 129 kb). [file 10143_2020_1399_MOESM1_ESM.pdf]

Impact of additional resection on new ischemic lesions and their clinical relevance after intraoperative 3 Tesla MRI in neuro-oncological surgery

Stefanos Voglis[1] MD, Timothy Müller[1] BSc, Christiaan H B van Niftrik[1] MD, Lazar Tosic[1] MD, Marian Christoph Neidert[1,2] MD, Luca Regli[1] MD, Oliver Bozinov[1,2*] MD*

[1] *Department of Neurosurgery and Clinical Neuroscience Center, University Hospital and University of Zurich, Frauenklinikstrasse 10, 8091 Zurich, Switzerland*

[2] *Department of Neurosurgery, Kantonsspital St. Gallen, Medical School St. Gallen, Rorschacher Strasse 95, 9007 St. Gallen, Switzerland*

*Corresponding author:

Stefanos Voglis, MD – stefanos.voglis@usz.ch

Department of Neurosurgery and Clinical Neuroscience Center

University Hospital and University of Zurich

Frauenklinkstrasse 10

8091 Zurich

Switzerland

Metastases primary tumor	No.
Lung carcinoma	6
Germ cell testicular tumor	1
Mamma carcinoma	1
Malignant melanoma	1
**Others**	
Epidermoid cyst	3
Central neurocytoma	2
Schwannoma	2
Meningioma	2
Chordoma	2
Glioneural tumor	2
Cavernoma	2
Radionecrosis	2
DNT	1
Echinococcus cyst	1
Myxoma	1
Neurinoma	1
PNET	1
Reactive gliosis	1

Supplementary Table 1 – Histopathological characteristics

DNT = dysembryoplastic neuroepithelial tumor; PNET = primitive neuroectodermal tumor (diagnosis before 2016 WHO classification of CNS tumor update)

			poMRI new infarct compared to ioMRI		
**Characteristics**	**No.**	**Distribution**	**yes**	**no**	**p-value**
**ioNM usage**					
yes	107	60.5%	68 (64%)	39 (36%)	*.283*
no	70	39.5%	38 (54%)	32 (46%)	
**Worsening of ioNM**					
yes	23	21.5%	13 (56.5%)	10 (43.5%)	*.585*
no	84	78.5%	55 (65.5%)	29 (34.5%)	
**Resection stopped b/c ioNM decline**					
yes	18	17%	12 (67%)	6 (33%)	*.974*
no	89	83%	56 (63%)	33 (37%)	

Supplementary Table 2 – Intraoperative neuromonitoring

Study population characteristics regarding usage and decline of intraoperative neuromonitoring. Significance levels were calculated using Pearson's chi-squared test. ioMRI = intraoperative MRI; poMRI = postoperative MRI; ioNM = intraoperative neuromonitoring; b/c = because; No. = number
