# Supplementary material for: Impact of additional resection on new ischemic lesions and their clinical relevance after intraoperative 3 Tesla MRI in neuro-oncological surgery
Source: Neurosurg Rev. 2020 Sep 30;44(4):2219–27. doi: 10.1007/s10143-020-01399-9 (PMC8338811; doi:10.1007/s10143-020-01399-9)
Supplement: Supplementary file 2 — (PDF 525 kb). [file 10143_2020_1399_MOESM2_ESM.pdf]

**Impact of additional resection on new ischemic lesions and their clinical relevance after intraoperative 3 Tesla MRI in neuro-oncological surgery**

*Stefanos Voglis[1*] MD, Timothy Müller[1] BSc, Christiaan H B van Niftrik[1] MD, Lazar Tosic[1] MD, Marian Christoph Neidert[1,2] MD, Luca Regli[1] MD, Oliver Bozinov[1,2*] MD*

[1] *Department of Neurosurgery and Clinical Neuroscience Center, University Hospital and University of Zurich, Frauenklinikstrasse 10, 8091 Zurich, Switzerland*

[2] *Department of Neurosurgery, Kantonsspital St. Gallen, Medical School St. Gallen, Rorschacher Strasse 95, 9007 St. Gallen, Switzerland*

*Corresponding author:

Stefanos Voglis, MD – stefanos.voglis@usz.ch

Department of Neurosurgery and Clinical Neuroscience Center

University Hospital and University of Zurich

Frauenklinkstrasse 10

8091 Zurich

Switzerland

# Supplementary Figure 1

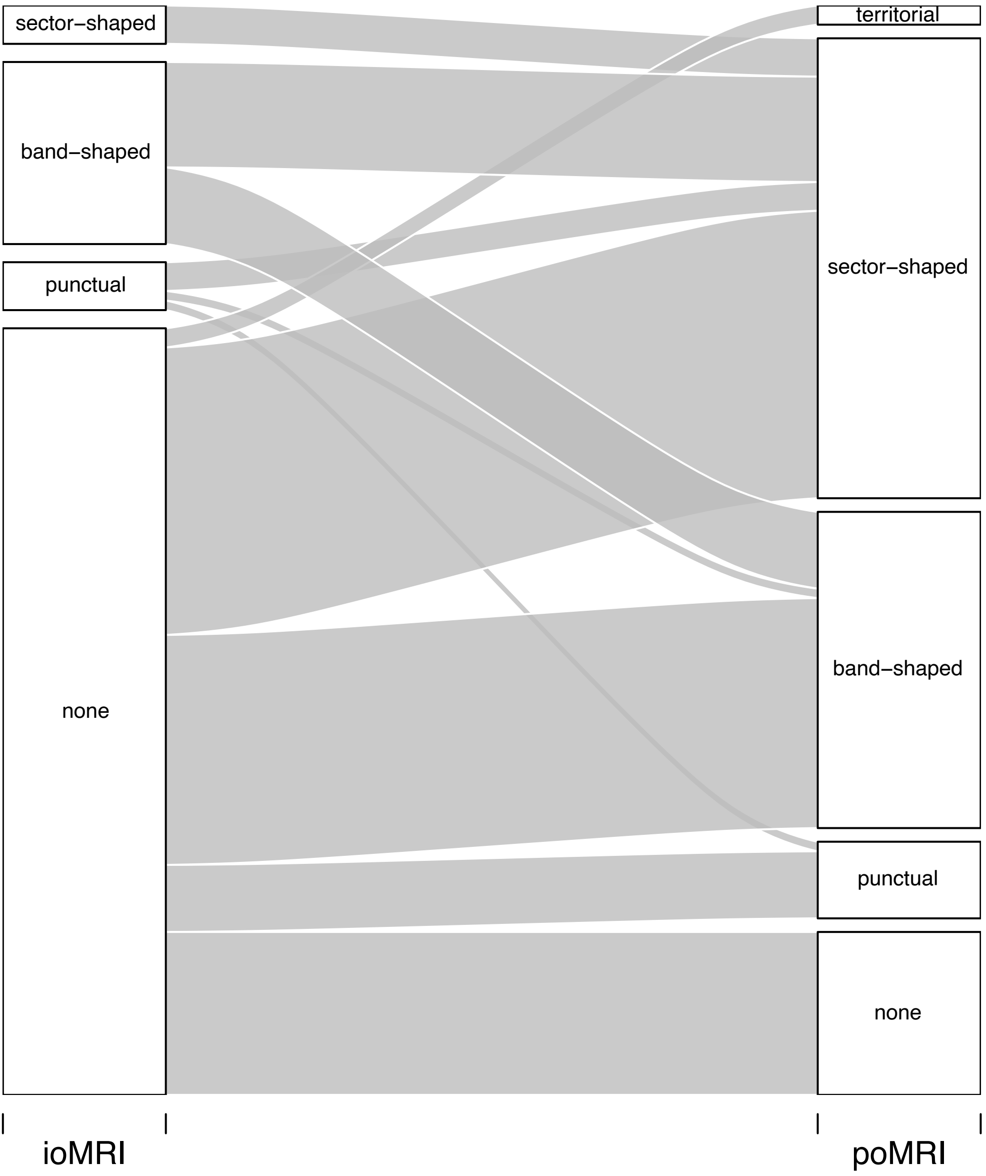


**Supplementary Figure 1:**
Evolution of highest infarct classes in postoperative (poMRI) vs. intraoperativeMRI (ioMRI) for matched cases with additional resection after ioMRI. (none = no infarct present)

# Supplementary Figure 2

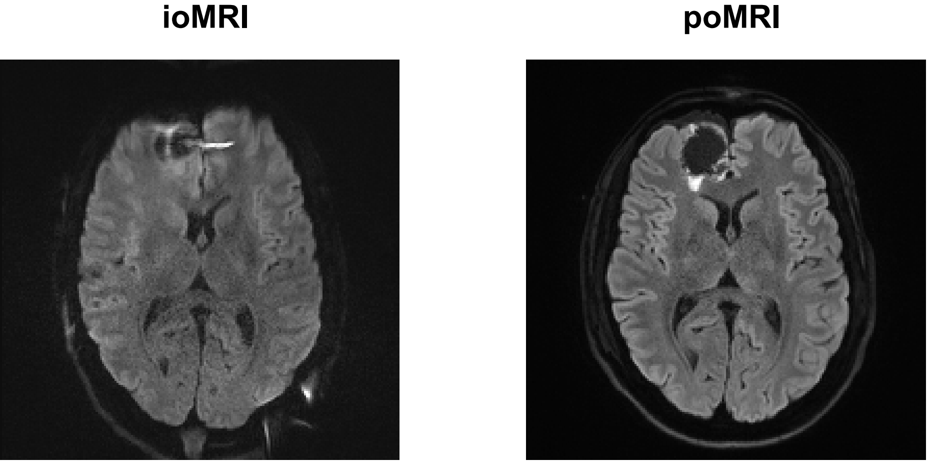


**Supplementary Figure 2:**
Illustrative case example:
33 y/o male with resection of a WHO grade III oligodendroglioma in F1. Intraoperative MRI (left) showed no signs of DWI restriction. However, after continued resection poMRI (right) showed a sector-shaped infarct at the posterior resection cavity. Postoperatively the patient showed no new neurological deficits.

## Supplementary Figure 3

**a**

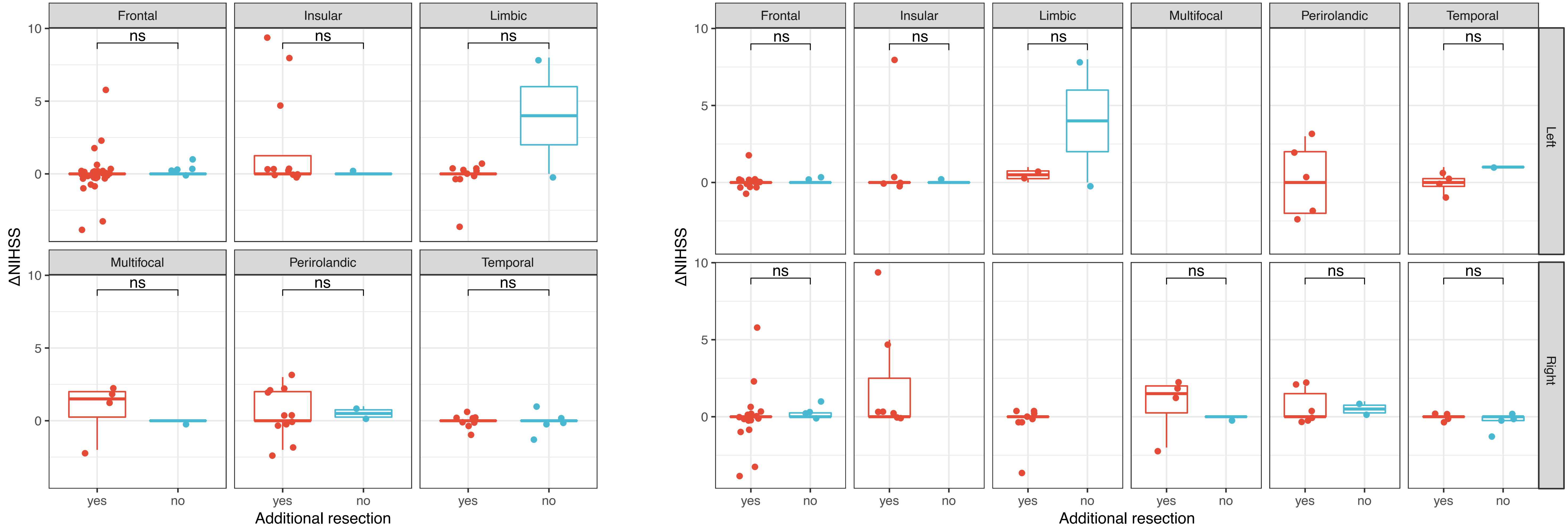


**b**

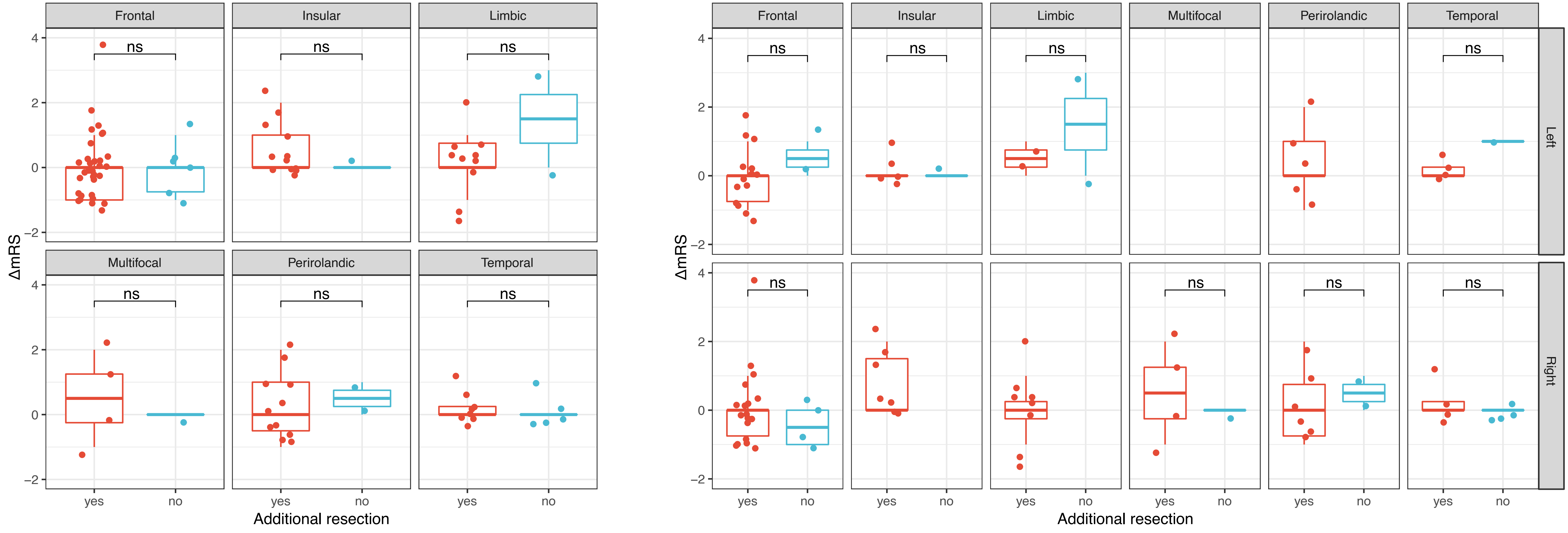


**Supplementary Figure 3:**
(a, b) Comparison of relative changes in NIHSS (a) and mRS (b) at discharge compared to admission for cases with (red) and without (blue) additional resection after intraoperative MRI. Left plots facetted by major anatomical localization, right plots by anatomical localization and lateralization; only cases with a new infarction on postoperative MRI are shown
